# Art box deliveries: The experiences of people with dementia and their carers during the Covid 19 lockdown

**DOI:** 10.1177/14713012221124863

**Published:** 2022-09-02

**Authors:** Christina Armstrong, Anji Archer, Valerie Critten, Sarah Critten

**Affiliations:** 4903 University of the Arts London, London, UK; 3769 University of Hertfordshire, Hatfield, UK; 5488 Open University, Milton Keynes, UK

**Keywords:** people with dementia, caregivers, Covid 19 lockdown, art activities, social communication and enrichment

## Abstract

Art workshops have been looked at before in terms of impact for people with dementia but never those conducted remotely during a pandemic lockdown. Two artists, working with local museums, provided Art workshops for people with dementia and their caregivers. Due to the first Covid 19 lockdown in the UK, the artists set up a weekly delivery service of Home Art Boxes to thirty-three people with dementia and their caregivers over a period spanning 11 months. The artists received funding from local organisations and the Community Lottery Fund. Thematic analysis of the feedback from the participants regarding the project and the artists themselves provided the data for this evaluation of the project. Seven main themes were identified: organisation of the project; community and connections; supporting the caregivers; enjoyment and enrichment; well-being and cognitive benefits of the projects; equipment and instructions; and drawbacks within the project. The participants’ feedback enabled the artists to improve the contents and instructions given each week so that they were able to adjust the activities for those people with dementia whose condition was declining. Implications are that remote Art workshops are possible during lockdown restrictions, but that personal communication is equally important.

## Introduction

Dementia is a neuropsychological group of disorders in which the brain gradually deteriorates and functions, such as those that rely on short term memory, decline over time (Braaten et al., 2006). It affects one in ten people over the age of sixty, and one in two over the age of ninety (Alm et al., 2007). Communication and social awareness begin to decline (Rousseaux, Sève, Vallet, Pasquier &Mackowiak-Cordoliani, 2010) and a person with dementia gradually loses their sense of themselves and their identity (Acton et al., 2007; Caddell & Clare, 2013). Many people with dementia exhibit psychological symptoms such as depression, agitation, apathy and aggression (National Collaborating Centre for Mental Health (NCCMH, UK. 2007). The consequence of having dementia affects people in almost every aspect of their lives and can be challenging not just for themselves but also for their family members and caregivers (Alm et al, 2007). Often, caregivers find that their relationship with the person with dementia changes as they have to assume more of the responsibilities for daily living. Many people with a spouse or partner with dementia try to maintain their relationship within a ‘sustained couplehood’ (Helstrom et al., 2007) but caregivers can be impacted by the behavioural disorders exhibited by people with dementia. This can lead to caregivers suffering mental health disorders such as anxiety or depression (Mazzi et al., 2020). It is very important that both people with dementia and their caregivers are assisted by medical health professionals and by other types of treatment or therapies such as group meetings and social contact, for example at Day Centres (Pongan et al., 2021) where activities include reminiscence to promote communication and enhance well-being and enrichment (Brooker & Woolley, 2007; Critten & Kucirkova, 2019).

At the beginning of 2020, items on the national news included reports from China of a new virus which was affecting people’s respiratory processes and was very contagious. There was speculation that the virus (a coronavirus) might spread outside of China and affect other countries. By March, alarm was raised that the coronavirus (now named Covid-19) was spreading to Europe and could spread to the UK, and a national lockdown was announced by the UK government to start on March 23rd (Department of Health and Social Care (DHSC), 2020). This meant that everybody, other than key workers, had to stay at home, and were only allowed out once a week to do vital shopping, or to take daily exercise (Lacobucci, 2020). As well as non-essential shops and businesses closing, all day centres and groups for the elderly and vulnerable also closed down as these groups were more likely to become seriously ill or die from the disease. Many people with dementia come into this category and they and their caregivers had to isolate at home, often with their support and care taken away (Brown, Mossabir, Harrison, Brundle, Smith & Clegg, 2021). Due to the decrease in support for people with dementia and their caregivers, this study evaluates a new approach to provide support remotely as there were concerns over the negative impact on well-being during the lockdown.

### The impact of isolation on people with dementia and their caregivers

People with dementia require complex therapies and treatment to reduce disordered behaviour. Due to the pandemic lockdown, many people were unable to continue with their therapy or meeting groups and this led to increased loneliness and isolation (Mazzi et al., 2020). A number of groups were able to continue online, but older people often do not have internet access, so this was not a possibility for them. Additionally, many paid caregivers who supported people with dementia stopped visiting, and members of their families or friends often became the only caregivers. This put great strain on the caregivers particularly in those circumstances where an elderly husband or wife had to look after their spouse (Mazzi et al, 2020). However, despite caregivers having to take more responsibility for their family members or friends, some people reported that they now had more time to be with them and to do more activities during the lockdown, albeit within the home environment (Brown et al, 2021).

### The importance of art-based activities for people with dementia

Art-based activities can take many forms such as 1-1 individual sessions with a client or in weekly group sessions, and is often organised by artists, therapists or by professional caregivers in a care setting (Chancellor et al., 2014). In the community, organisations such as museums have often provided boxes of artefacts to groups, day centres and care homes for use as reminiscence therapy, which has been shown to help people with dementia with communication and social contact (Flatt et al., 2015; MuseumNext, 2021). Quantitative measures such as psychological well-being tests have shown an increase in participant’s well-being and happiness levels during these types of sessions (Howarth, 2018).

Museums have also been at the forefront in providing workshop sessions for groups of people with dementia in which artefacts or artworks provide inspiration for art and craft activities, often leading to exhibitions of work, the creation of booklets, or short films being shown online (Armstrong & Archer, 2021). The benefits of such programmes also extend to their caregivers, as people with dementia increased their communication and reminiscence with them as well as increasing their art and craft skills (MuseumNext, 2021).

Although there is a limited amount of evidence on the efficacy of therapies (Beard, 2012), some of the findings suggest that engagement with art may help to decrease adverse behaviours and enrich the lives of people with dementia (Chancellor et al., 2014). Research findings of groups such as those by Rusted, Shepherd, & Waller (2006) suggest that over 40 weeks of therapy an art group showed improvements in ‘*aspects of mental alertness, sociability, physical and social engagement in clients with moderate and severe dementia’* (pp. 531) in comparison to a control group which showed deterioration in their behaviours. However, the control group were given activities which were normally available to them in their settings and were given no Art and Craft activities and no therapeutic approaches were utilised.

### Rationale for the study

The first two authors, who are both artists, became involved in art projects for people with dementia and their caregivers, and together they ran art workshops in conjunction with local museums. The projects were funded by different bodies including the Arts Council and local councils and organisations.

**Figure 1.**
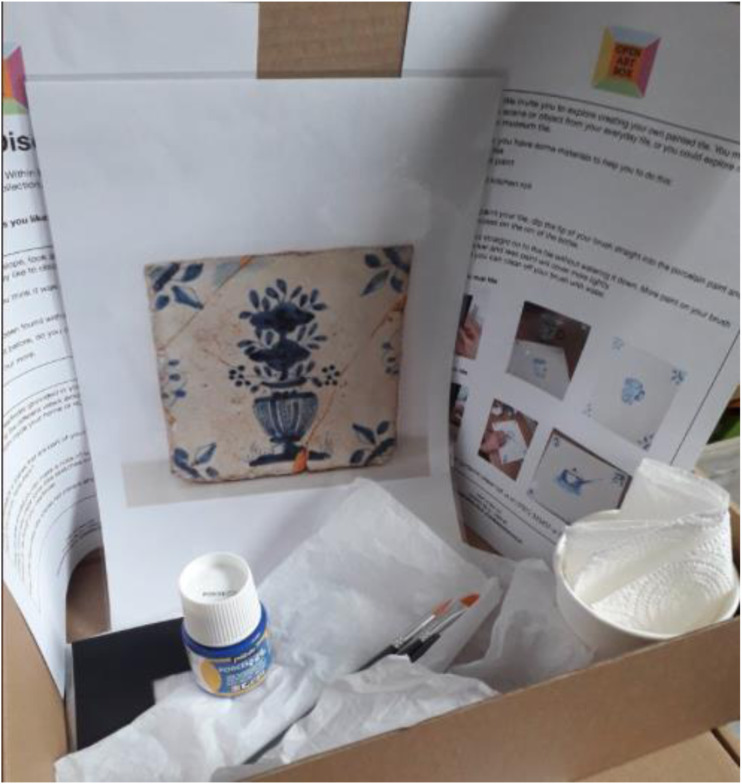
Contents of one of the art boxes showing a picture of a Victorian tile; some paint and brushes and paper; and instructions for that week’s activity.

**Figure 2.**
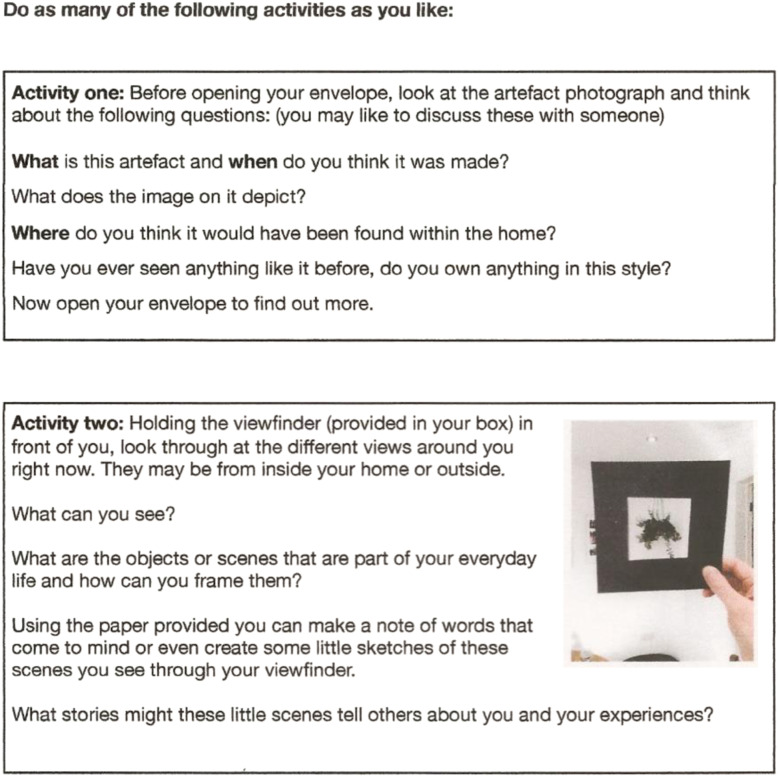
Page 1 of instructions given in the box illustrated in Figure 1.

**Figure 3.**
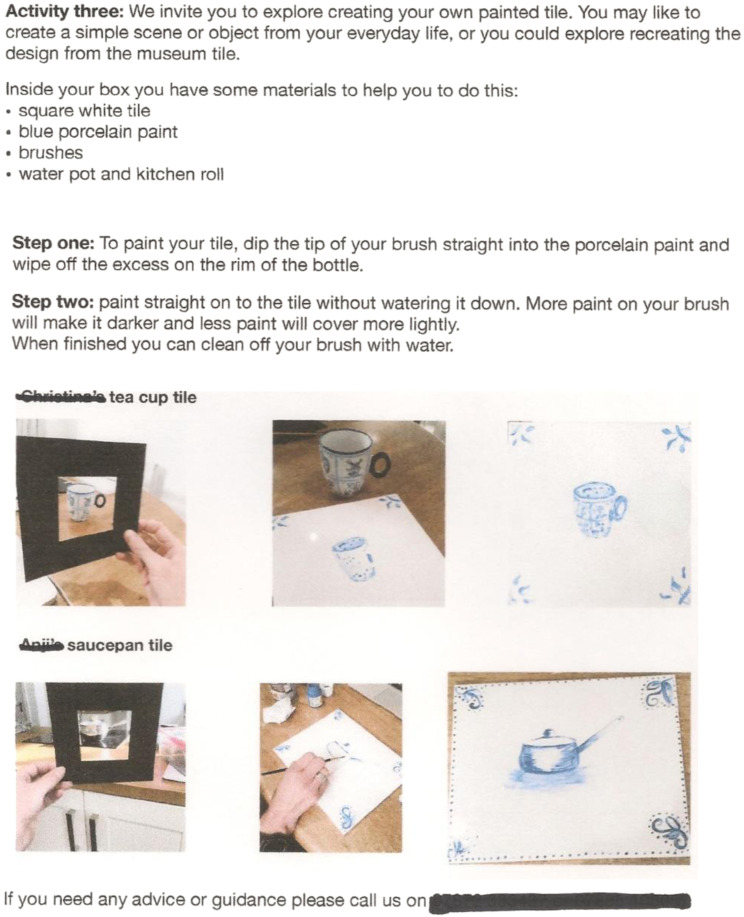
Page 2 of the instructions given in the box in Figure 1.

When the lockdown started in March 2020, the artists were running a programme of taster workshops for people with dementia and their caregivers in local community groups but had to finish because of government guidelines. The artists were very concerned for the well-being of their participants and telephoned each week during the earliest part of the lockdown. They decided to develop a project that adapted to the restrictions that were in place. Instead of the participants going to the workshops, the artists would deliver art activities in boxes each week to the participant’s homes. The boxes contained artefacts from two local museums as a central focus, e.g., Victorian tiles, old Valentine cards, or Art and Craft poster designs (see Figure 1) with relevant art materials and instructions to complete the tasks (see Table 1, and Figures 2 and 3).

**Table 1.** Contents of the art box in Figure 1.

Contents of the home art box
Information about Delft blue tiles from The Netherlands
Instructions of activities (two pages, see Figures 2 and 3)
Paper cup, paint brushes, blue paint, and kitchen paper tissue
Large picture of a blue and white tile
Viewfinder

Two projects were developed, one involving sixteen participants (running for 4 months), and another later one for twenty-nine participants (running for 5 months), under an umbrella term (for the purpose of anonymity) of Home Art Boxes. The artists were funded by local councils, local organisations and the Community Lottery Fund, and the two projects ran over a span of 11 months delivering boxes to thirty-three households in two towns and their surrounding areas. The gap in time after the first project was to allow the artists to fund-raise for the second project and to gather further materials. Table 2 illustrates some of the projects designed by the artists.

**Table 2.** Projects designed by the artists for the home art boxes.

Week	Phase 1: Oct–Dec 2020	Focus of the project
1	Roman nursing Figurine (Dea nutrix) from AD150	Photograph a treasured item from your house
2	Delftware tile (See Figures 1–3))	Produce own design of blue and white tile using paint provided
3	Victorian silhouette memorial card	Create a Victorian silhouette portrait from photo (or stencil provided)
4	WWI pastel drawing of soldiers in the Trenches by neville Lytton 1914-18	Make a felt poppy out of pre-cut pieces
5	C19th watercolour of Ashwell by samuel Lucas senior	Paint a watercolour landscape
6	C19th perks lavender Label from Hitchin lavender	Stitch a lavender design on a scented Lavender bag
7	Guide to the garden city (cover design) by Thomas Adams 1906	Produce a poster design using collage and tracing
8	Clockwork toy train 1870–1900	Produce a toy dancing figure from pre-cut pieces using split-pins
Week	Phase 2: Jan–Mar 2021	
1	Handmade tea caddy made by French prisoner of war, 1793–1815	Make a quilled design using pre-prepared quills
2	Late neolithic collared Urn (2500–2300 BC)	Produce a clay pot with air drying clay
3	Hitchin town football club pennant flag 1958	Design own football pennant in triangular form using fabric pens
4	Spitalfields silk formal dress C.1730–1750	Produce and decorate a handheld fan from paper and lolly sticks from pre-prepared pieces
5	Victorian Valentine’s Card	Design a card using lace, pictures and decorations provided
6	John Ogilby’s strip map 1698	Map a local journey, place or room using paper, pens and colouring pencils
7	Handmade playing cards C17th	Design your own playing cards using stickers on blank cards
8	Elizabeth Impey’s suffragette rosette badge 1907–14	Produce a suffragette rosette from crepe paper and card

As part of the funding process, the artists had to evaluate the two projects and obtain feedback from the participants involved in Home Art Boxes. This study amasses all of that information and evaluates the project from the experiences of the recipients of the Home Art Boxes and the artists themselves.

This project was innovative in design due to the need for remote workshops during the pandemic which have not previously been examined in relation to their impact in people with dementia or their caregivers. The research consists of an evaluation of existing secondary data collected from remote workshops in the form of the delivery of art boxes during the Covid 19 pandemic in 2020. The deliveries were designed to help reduce isolation, maintain social contact and improve well-being in people with dementia and their caregivers.

The research questions focused on two main themes:

1. What were the experiences for people with dementia and their caregivers and how have the Home Art Boxes helped them during the Covid 19 lockdown?
2. What were the successes of the Home Art Boxes and what changes need to be made for the next project?

## Methodology

### The participants

The participants (See Table 3) were enlisted from a number of sources: people already attending local dementia groups; self-referred people with dementia and/or caregivers through local advertising; or through local care agencies. The people with dementia were not all formerly diagnosed with dementia but had significant memory loss which interfered with their daily living. Because the participants with dementia would need to be able to work on the projects independently, or just with a caregiver, they were generally in the mild to moderate stages of dementia.

**Table 3.** Information about the participants in the home art box project.

Participants	Gender	Ages
People with dementia	9 males	6 young onset (under 65)
	9 females	12 over 65
Caregivers	4 males	4 aged under 65
	11 females	11 over 65

### Ethics

Although the data contained in this evaluation is secondary, and consent forms had been completed by the participants of Home Art Boxes, it was necessary to gain further consent for the data to be used for a research study. Approval for this study was given by the Open University HREC panel, no HREC/3942/Critten.

### Measures and procedures

Three evaluations of the projects were collected from people with dementia and caregivers and the artists themselves, and categorised by the researchers into a themed analysis:

1. Questionnaires halfway through and at the end of the projects. These consisted of questions regarding the impact of the deliveries: the enjoyment of the tasks; the quality of the equipment and the instructions; the impact on their wellbeing and creativity, and interest in further development of their skills.
2. Weekly feedback by the participants in each box which asked: If you have any comments about this box or the project overall, we always welcome your feedback, so please use the space below if you would like to share experiences or comments. This was to find out if the individual tasks worked for the participants, were they suitable and manageable.
3. Evaluations were written from field notes and after discussions with the artists.

### Data analysis

All the feedback from the participants was collected and a themed analysis of the data produced seven main themes: Organisation of the project; Community and connections; Supporting the caregivers; Enjoyment and enrichment; Well-being and cognitive benefits of the project; Equipment and instructions; Drawbacks within the project.

## Findings

### Organisation of the project

Once the lockdown started, the artists realized that many local services for people with dementia, such as group meetings and social clubs, had stopped, and that many of the participants of the workshops were alone and lacking support.

*“With the arrival of the pandemic, we became unable to continue with any of our plans, but we were still in contact with all the participants from our workshops, who we realised were extremely vulnerable and particularly isolated in the situation. We supported them with weekly phone calls but wondered what more we could do.”*

In response they decided to set up an Art Box delivery service, by delivering materials from the workshops to the participants at their homes. They decided to fund raise to enable this to continue:

*“It is a hugely time-consuming way to run a project and we did struggle sometimes to get all the planning, instructions and materials sourced and prepared in time each week - this became even more of a problem when shops were closed during lockdown.”*

### Community and connections

When the artists delivered the boxes, they found that many people wanted to talk to them at the time of delivery, and the pick-up of the previous week’s boxes. The artists ensured that they held a conversation with the participants, and this often took extra time,

*“We found that due to the level of need and isolation, some participants required much more time than we had allowed. (15 mins per visit sometimes became 45 mins).*

However, this contact was extremely important for maintaining communication. One of their participants with dementia said: “*Helping me stay in touch, and in contact as I feel like a prisoner…. The regular contact has been important”.* Another participant with dementia said: *“I really enjoy the chat at the door each week”*; and maintaining a conversation with others either about themselves or about the art activities in the boxes helped many people with dementia as commented by one of the caregivers:

*“She particularly enjoys that it comes to the doorstep and that she has a chance to chat once a week with someone who shows an interest in her work and skills.”*

Others liked that their work would be displayed both on social media and on the artists’ website. One participant with dementia commented: *“I feel more connected because I know what I do is put together with other people and they have their own ideas.”* In addition, another participant with dementia said that it gave them something to talk about with families or friends:

*“Others that have come to my house have commented on the things I have been doing. Gives us things to talk about and has been the start of conversations. I have also talked to daughters and Crossroads staff about the boxes”*

### Supporting the caregivers

The art activities are not just important for people with dementia but for the caregivers too, to help them maintain contact within the community. One caregiver commented:

*“I have felt less isolated when other things have stopped and there has been a greater burden on the carer. It has provided something that I have not had to organise and taken a weight off my shoulders.”*

Many programmes for people with dementia such as in clubs and workshops only involve the person with dementia to enable caregivers to have respite, however, this project usually involved both people with dementia and their caregivers except in certain circumstances. The artists commented:

*“We had other situations that the caregiver saw the activity as only for the person with dementia and chose not to get involved. In those instances, caregivers saw it as a chance for their loved one to have something just for them, independent from the carer. We respected this decision too and in the right situation saw it work very well, giving a level of independence to person with dementia. ‘Something just for them’.”*

Occasionally, the caregiver was the person who wanted to engage with the activities rather than the person with dementia and the artists supported this as they felt that their needs were just as important:

*“We felt strongly that supporting the carer was an equally important part of supporting the person with dementia too and the feedback from that has suggested this.”*

### Enjoyment and enrichment

The aim of this project was to improve and enrich the lives of both people with dementia and their caregivers during the isolation engendered by the pandemic, particularly those people with moderate to severe memory loss who may ‘live in the moment’. Several of the caregivers commented on how the art activities had helped their family members to maintain or even restore some of the cognitive functions lost previously:

*“She had recently completely stopped doing her craft projects as she could not self-motivate / organise her skills / remember what she was doing.* (This project) *came along just at the right time when she still has the comprehension to do some of the tasks unsupervised, or when confused or forgetful, could still enjoy doing them with us.”*

This was supported by another caregiver who said:

*“P’s lifelong capacity to do art and craft has diminished with alarming speed over the last couple of years, so it is wonderful to have this instead and it certainly makes her feel involved and confident.”*

Indeed, many people with dementia and caregivers said that they would like the delivery of the art activities to continue even if the lockdown was discontinued. One caregiver said:

*“Even if groups were allowed to meet up, I think this can offer some other advantages to those who spend a lot of time at home with caregivers - structure / inspiration and being able to share absorbing tasks, for instance.”*

### Well-being and cognitive benefits of the projects

In this project, people with dementia commented on how engaging with the project had helped their mental health saying, *“I have depression and anxiety, so the box allows me to be involved in activities that give me focus on a task. I get into the activities which feels really good for me.”* A carer also commented about the person with dementia that she looks after: “*She was a bit agitated that day and it helped calm her.”*

Indeed, over the length of the several months of the delivery of the work boxes, if there were signs of declining mental health and/or decreasing cognitive abilities of their clients with dementia, the artists were prompted to further consider the activities they provided:

*“Due to dementia being a degenerative disease (and the added effect of the pandemic and isolation), we have witnessed a decline in some of our participants, that has meant we have had to adapt the way the activity is delivered, simplifying it down, reducing the amount of text and language used and decreasing the number of ‘steps’ to an activity.”*

The feedback from caregivers and people with dementia seemed to indicate that many of the clients improved during the work box activities. This suggests that the artists were able to adjust to the needs of their clients very quickly in order to keep their interest.

Although the artists had not initially considered the benefits of reminiscence when starting the project, caregivers mentioned how some activities inspired reminiscences which both carer and the person with dementia could share and enjoy:

*“We both enjoyed seeing this week’s artefact of the painting of Rook’s Nest and learning a bit about its history. This enabled us to reminisce about walks we have taken over the past 40* *years past the property.”*

The benefits of using resources from local museums meant that many people were inspired by photographs of artefacts in their Art Boxes even if they could not enjoy handling the artefacts themselves. One of the caregivers mentioned how she enjoyed the toy workshop inspired by a 19^th^ century toy train from the local museum. She said, *“I really enjoyed this project, thinking about my childhood days playing with these things, also thinking about the friends who played with me. We are all in our 80s now. This project certainly helped me to reminisce.”*

### Equipment and instructions

This was the area in which there was a mixed response by the participants. While all the participants loved getting the boxes, a number of them found some of the tasks either too complex or the equipment difficult to manage or the instructions were a bit too complicated. Regarding the equipment in the boxes one person with dementia said:

*“I really look forward to the box arriving. As soon as the box comes, I grab it, it’s got all the right equipment and there are always really nice things prepared for me to do. I get stuck into every project.” *A caregiver agreed with this by saying: *“The equipment is good, and the instructions are clear, about the right amount and not too confusing.”*

However, some of the participants found that some of the activities were too difficult; one caregiver reported that “*For us this was too involved, and we lost interest. For example, the poem under the picture seemed far too remote for us to grasp.”* Another of the caregivers felt that the task was too involved for their family member to manage:

*“He did a rough sketch but unable to transfer it to the main paper. He found it difficult to transfer his thoughts to paper. … On reflection I should have given him ‘highlights’ i.e., our house and got him to draw a route to the post box on our estate then added to it.”*

Other caregivers thought that the equipment was too difficult for their family member. One reported that: “*She had never used charcoal before so took a bit of time to understand how to use it, but she had fun with it and spent a while doing it.”* Another caregiver said:

*“Mum was good with her colour selection in attempting to replicate the town centre image but was less inclined to complete the image. I think she found it a bit daunting.”*

Other people with dementia enjoyed doing the activities but found that their physical abilities sometimes limited them, for example one caregiver reported, “*Unfortunately we found we could not do this project.* (family member’s) *eyesight limits him, and I found it difficult to adapt it for us.*’” Another caregiver said that “*We loved the activity of quilling, just had a problem with the PVA glue as it wouldn’t come out very well. It hurt my hands squeezing the bottle just to get a little out.”*

Despite some of these difficulties, caregivers thought that the activities were worthwhile even if the results were not what they had intended:

*“This was a difficult project and did take a long time, but he did achieve the colouring. Assembling the puppet was beyond his capabilities and I am not able to ‘work’ the puppet, but we were pleased with the end result.”*

Additionally, problems with short term memory affected the abilities of some people with dementia to remember their enjoyment of completing a task. One caregiver commented:

*“She doesn't necessarily remember each task, but then she sees some of the objects around her that she has created and feels happy and proud. Without these weekly boxes we would probably do the odd thing with her, but not nearly so much or as varied.”*

### Some drawbacks within the project

One consideration of the artists was whether they could keep in close contact with people with dementia if they did not live with their caregiver, as communication between artists and participants is considered so important:

*“*(We) *had a participant who was deaf, although she could lip read, I could not contact her easily between visits and had to rely on her daughter to find out how things were going. There were often times I could not get hold of her daughter and I found the participant was not feeling motivated to do the activities. These boxes seem to work particularly well when there is good communication with the family, and they are supportive and interested.”*

Another difficulty with the ‘remote’ aspect of the workshops was that the participants sometimes were unable to complete the activities for some reason. The artists felt that if they had been together in a workshop situation, they would have been able to help and encourage the completion of the tasks. The artists said:

*“Our inability to be physically present as they attempted the activities meant occasionally participants lost confidence and stopped with an activity. We offered telephone support, however there were very few occasions that they would ring us for help. I think they didn’t want to trouble us, (despite reiterating the message that that’s what we put our numbers on there for).”*

The artists also found that they had some difficulties in running the projects:

*“I think occasionally we felt a little overwhelmed by the emotional toll of trying to support people through such challenging times. Two of our participants died over the course of the project, which was particularly upsetting.”*

Despite these difficulties and drawbacks, the artists felt that the projects had helped many of the participants to get through the lockdowns. One of the artists commented:

*“It has been a learning journey, which has had its challenges on many levels, emotionally and physically. The wonderful part of being involved in a project of this nature has been the joy to see people smiling, even if they haven’t been able to complete their artwork. By delivering the boxes we offered a human touch as well as a creative one. Most of the participants are looking forward to the next round and to see what it will offer, so that is very exciting.”*

## Discussion

There were two main research questions regarding this project, which consisted of an evaluation on existing secondary data. The first question considers the experiences of Home Art Box deliveries for people with dementia and their caregivers during the lockdowns due to the Covid 19 pandemic 2020–2021 (DHSC, 2020).

The participants were in favour of the project and reported that seeing the artists and speaking to them helped them to maintain social contact as the participants often felt isolated and lonely. This was similar to findings by Mazzi et al., (2020) who reported that their participants had times when they felt alone and disconnected as they were often deprived of care services. The doorstep contact was considered an important part of the project as the artists felt that it increased the well-being of the participants including caregivers. Pongan et al., (2021) suggested that behavioural disorders increased in those with dementia during the lockdown and caregivers experienced poorer mental health as a result. The delivery of the Art Boxes helped the participants to maintain communication and may have helped to reduce the feeling that they were living in ‘shrinking worlds’ as suggested by Talbot and Briggs (2021). The caregivers reported that the activities diverted people with dementia when they felt agitated or felt low and disinclined to do anything (see NCCMH, 2007) and improved the caregivers’ own mental health when they felt anxious or depressed by the lockdown and these results duplicated those of Mazzi et al., (2020). People with dementia reported that they appreciated the contents of the Art Boxes as the activities helped to occupy them during the pandemic and gave them a topic to communicate with their family and friends (see also Acton et al., 2007).

The art activities were not just important for the people with dementia, but also for the caregivers too to keep them in contact within the community, and these results confirmed the findings of Alm et al., (2007). Many programmes for people with dementia such as in clubs and workshops only involve the person with dementia to enable caregivers to have respite (see Critten & Kucirkova, 2019), however this project involved both, as the artists believed that the caregivers’ needs were equally as important (see Mazzi et al., 2020).

Although there is some doubt as to whether Art therapies and activities provide long-lasting cognitive benefits or improve the condition of dementia suggested by Beard (2012), some studies report cumulative changes in the responsiveness of their participants particularly in the areas of mental acuity and physical engagement in the activities (Rusted et al, 2006). Research into Art Museum engagement reported benefits such as social cohesion, mental stimulation and an increase in self-esteem by people with dementia and their caregivers (Flatt et al, 2015; Howarth, 2018). The aim of this project was to improve and enrich the lives of both people with dementia and their caregivers during the pandemic, particularly those people with moderate to severe memory loss who may ‘live in the moment’ (see Stopford et al, 2012). Overall, the participants found that the activities were inspiring to complete and a number of them evoked reminiscence which has been shown to increase wellbeing in people with dementia, and this study confirmed the findings by Alm et al., (2007). Although the artists had not initially considered the benefits of reminiscence while starting the project (see Critten & Kucirkova, 2019), caregivers mentioned how some activities inspired reminiscences which both carer and the person with dementia could share and enjoy. The benefits of using resources from local museums meant that many people were inspired by photographs of artefacts in their Art Boxes even if they could not enjoy handling the artefacts themselves. Flatt et al., (2015) also reported that the participants in their study particularly enjoyed ‘hands-on’ activities after reminiscing about artefacts seen in a museum.

However, some of the activities proved too difficult for the people with dementia, either because they were unable to follow the instructions or because they were unable to manipulate the equipment provided (see Braaten et al., 2006 for descriptions of dementia), and research by Flatt et al., (2015) suggests the need for customising the activities according to the disease stage of the people with dementia. Some of the caregivers were able to adjust the activities to suit the person with dementia, but this relied on the caregivers being present at the time and some of the caregivers did not live with their family members. Over the length of the several months of the delivery of the work boxes, if there were signs of declining mental health and/or decreasing cognitive abilities of their clients with dementia, the artists were prompted to further consider the activities they provided. Flatt et al., (2015) also considered this an issue in their study with one participant suggesting that the activities should be geared for the older members of the group.

The second question concerned the evaluations of the project made by the artists. The project involved a large amount of administration at first to enable funding to be raised for the project (see Van Donk & Molloy, 2008). Each part of the project had to be itemised in the applications. One of the main requirements reported by studies involving Art activities described the need for the artists or other managers (“the locksmith”, Brooker & Woolley, 2007) to lead the activities, and thus ensure the continued engagement and enjoyment of people with dementia. Some of the difficulties was because of the length of time it took to prepare and deliver the boxes as the artists had not realised the length of time some of the participants needed to talk on the doorstep. Another problem was finding the equipment they needed when many shops were closed. They were able to order online but there were sometimes delays in their deliveries.

The artists felt that one of the biggest challenges was being able to cater for the needs of the people with dementia because of their decreasing cognitive and physical abilities in comparison to the other participants (see Braaten et al, 2006). Before the pandemic, the artists provided workshops for people with dementia which normally were 6 weeks in length. The Home Art Box projects were over the course of several months. This led to them having to provide activities especially tailored to their needs to enable them to continue in the project. The artists got to know the participants very well and were very sad when two of them died. However, both artists felt that the projects had been worthwhile in enriching the lives of the participants because delivering the boxes meant that they maintained a personal contact and enabled social communication, albeit limited, as well as the creative aspect of the Home Art Boxes.

### Implications

The first implication from this evaluation of the Art Boxes project is that remote workshops in this form of home delivery are a viable option for supporting people with dementia and their caregivers at home and may have a positive impact on well-being especially during a pandemic situation. To our knowledge this is a novel finding, and we hope will generate further research into why this might be the case. The themed analysis suggests two key processes that were beneficial for participants and could be explored further, i.e., reducing isolation and increasing social contact/communication and the enrichment and enjoyment provided by the art activities. Further quantitative examination could explore the relationship between these two processes and measures of well-being more conclusively.

The feedback from the participants and the artists’ own evaluations from the project have enabled the artists to plan future projects both involving workshops with the museums as before, but also continuing the delivery of Home Art Boxes as they have been so well received. The practical implications of the original project will be implemented in the design for future projects, i.e., how much time to allow for visiting participants and altering or removing activities that may have been less manageable for participants. The need for the remote workshops remains as the pandemic continues and many older people still feel vulnerable in socialising with others outside of their homes.

Another outcome of the Home Art Box deliveries project has been interest from two independent private living apartments. The landlords felt that there is further scope in the future for in-house art workshops for residents to attend. This was a result from requesting authorisation for photos and filming to take place on their premises and conversations with a duty manager.

### Limitations and future directions

This project developed solely as a result of the lockdown during a pandemic. There has been no previous research into this area for people with dementia and their caregivers. Further research would be welcomed into this new area of research, for example, a comparison of levels of well-being in face-to-face versus remote Art workshops. Another aspect that might need clarifying is how ‘remote’ the Art Boxes project really were given that the artists maintained social contact with the participants, however the artists were not present when the Art activities took place.

## Conclusion

The Home Art Boxes project was developed by two artists after their workshops for people with dementia and their caregivers were stopped due to the Covid 19 pandemic. Evaluations and feedback from the participants illustrated how much enjoyment they felt both because of the social interactions with the artists, and because of the enrichment activities offered by the Art Boxes. The artists had to continually monitor the feedback to ensure that they provided suitable activities for the participants, particularly for those whose condition was deteriorating. The evaluations also provided information for future projects designed by the artists to support the well-being of people with dementia and their caregivers.
